# Use of genomics to explore AMR persistence in an outdoor pig farm with low antimicrobial usage

**DOI:** 10.1099/mgen.0.000782

**Published:** 2022-03-28

**Authors:** Nathaniel Storey, Shaun Cawthraw, Olivia Turner, Margherita Rambaldi, Fabrizio Lemma, Robert Horton, Luke Randall, Nicholas A. Duggett, Manal AbuOun, Francesca Martelli, Muna F. Anjum

**Affiliations:** ^1^​ Animal and Plant Health Agency, Weybridge, New Haw, Addlestone, Surrey KT15 3NB, UK; ^2^​ Great Ormond Street Hospital for Children, London WC1N 3JH, UK; ^3^​ University of Bologna, Via Zamboni, 33, 40126 Bologna BO, Italy; ^4^​ Teeside University, Campus Heart, Middlesbrough TS1 3BX, UK

**Keywords:** pigs, antimicrobial resistance, antimicrobial usage, wild birds, seagull, heavy metal resistance

## Abstract

Food animals may be reservoirs of antimicrobial resistance (AMR) passing through the food chain, but little is known about AMR prevalence in bacteria when selective pressure from antimicrobials is low or absent. We monitored antimicrobial-resistant *Escherichia coli* over 1 year in a UK outdoor pig farm with low antimicrobial usage (AMU) compared to conventional pig farms in the United Kingdom. Short and selected long-read whole-genome sequencing (WGS) was performed to identify AMR genes, phylogeny and mobile elements in 385 *E. coli* isolates purified mainly from pig and some seagull faeces. Generally, low levels of antimicrobial-resistant *E. coli* were present, probably due to low AMU. Those present were likely to be multi-drug resistant (MDR) and belonging to particular Sequence Types (STs) such as ST744, ST88 or ST44, with shared clones (<14 Single Nucleotide Polymorphisms (SNPs) apart) isolated from different time points indicating epidemiological linkage within pigs of different ages, and between pig and the wild bird faeces. Although importance of horizontal transmission of AMR is well established, there was limited evidence of plasmid-mediated dissemination between different STs. Non-conjugable MDR plasmids or large AMR gene-bearing transposons were stably integrated within the chromosome and remained associated with particular STs/clones over the time period sampled. Heavy metal resistance genes were also detected within some genetic elements. This study highlights that although low levels of antimicrobial-resistant *E. coli* correlates with low AMU, a basal level of MDR *E. coli* can still persist on farm potentially due to transmission and recycling of particular clones within different pig groups. Environmental factors such as wild birds and heavy metal contaminants may also play important roles in the recycling and dissemination, and hence enabling persistence of MDR *E. coli *. All such factors need to be considered as any rise in AMU on low usage farms, could in future, result in a significant increase in their AMR burden.

## Impact Statement

The rise in antimicrobial resistance is of concern globally. In this study we demonstrated that when selective pressure from antimicrobials are low in the farm environment, it significantly reduces the overall burden of MDR *E. coli *. However, presence of a low basal level of MDR *E. coli* over the time period sampled indicated persistence of certain clones. Although the cause for persistence was not clearly identified we can speculate that selective pressure from other agents such as heavy metal or recycling of clones through dissemination between different pig groups and birds leads to on-farm persistence, which require further investigation in future.

## Data Summary

The authors confirm all supporting data and protocols have been provided within the article or through supplementary data files.

## Introduction

Antimicrobial resistance (AMR) is widely recognised as an urgent global health threat, with high levels of resistance reported in community-acquired and health-care associated infections from all regions providing surveillance data to the World Health Organisation [1]. To help mitigate the risk posed by AMR in the United Kingdom (UK), the recent UK Government’s 5 year national action plan on AMR has the One Health approach at its core [2], which recognises that the health of animals, humans and the environment are intrinsically linked. In the European Union, EU member states have adopted a wide range of strategies and surveillance activities concerning reducing the use of antimicrobials in humans and food production. Although assessing the impact of this reduction is difficult, a reduction in antimicrobial usage (AMU) has been linked to a reduction in AMR in humans and livestock from both national surveillance as well as specific research activities [3, 4].

While antimicrobial use in pigs is predicted in future to increase globally, there has been considerable effort and success in reducing usage in the UK [5–7]. Within the UK, even though pigs have the highest antimicrobial usage among major food-producing animals (110 mg kg^−1^ in 2018); usage has decreased by 60 % compared with 2015 [5]. Previous studies on AMR in *E. coli* isolated from pigs on farms that use antimicrobials for therapeutic purposes have highlighted the importance of plasmids and horizontal transmission of AMR genes [8–10]. Mobile genetic elements (MGEs) such as plasmids, insertion sequences, transposons and integrons are thought to be the main drivers of horizontal transmission and acquisition of AMR genes [11–13].

The importance of AMR in wildlife specifically in relation to synanthropes, that is animals living within and benefiting from close proximity to humans, is becoming increasingly evident [14–16]. This is of particular note in the transfer (in either direction) of zoonotic or commensal bacteria between wildlife reservoirs and food producing animals on farms or at processors [17–19]. In previous studies, seagulls have been shown to be carriers of MDR and extended-spectrum beta-lactamase (ESBL) producing *E. coli* as well as other pathogenic Enterobacteriaceae and may act as important environmental reservoirs or transporters of resistance genes and zoonotic bacteria [20–25].

There has been limited work investigating the prevalence of MDR *E. coli* in livestock, and dissemination in the farm environment in the absence of selective pressure from antimicrobials. For this purpose, an outdoor pig farm which compared to a conventional pig farm, has a history of low AMU (10 mg kg^−1^; see Methods) for more than 5 years due to individual treatment of clinically diseased pigs, was selected. Wild birds, and in particular seagulls, were observed on the farm’s premises, and both livestock and gull faecal samples, where available, were included to explore the potential contribution of both to the AMR burden. Detailed genomic analysis of isolates were performed in order to explore the tenacity of AMR genes, and the diversity in the *E. coli* that harbour them at the isolate and MGE level, when selective pressure from AMU is low.

## Methods

### Faecal sampling and bacterial preparations

Pig faecal samples were taken from a low AMU, high-welfare, outdoor UK farm comprising a large breeding site with a connected nursery and finishing site. The antibiotic usage in this farm (~10 mg kg^−1^) was more than ten times lower of the national UK average usage in pig farms recorded at the same time across UK (131 mg kg^−1^) (https://www.gov.uk/government/publications/veterinary-antimicrobial-resistance-and-sales-surveillance-2018). Samples were collected at three time points during 2017–2018: T1, T2, T3 (0, +6, +12 months respectively). Five different age classes of healthy pigs were sampled; the brief description and age range for each is: weaners (piglets; 4–10 weeks old), gilts (female pigs/sows yet to produce litter; ~6 months old), farrowing (lactating sows; 9 months to 2–3 years), dry sows (gestating sows/sows awaiting service; 9 months to 2–3 years), and grower/finishers (adult pigs; growers: 10–14 weeks, finishers: >14 weeks). Age classes were kept in separate areas of the farm. Sixty individual fresh faecal samples per available age class were collected at each time point. Not all age classes could be sampled at all time points: for T1 and T3 samples were taken from farrowing, gilt, dry, grower/finisher and weaner age classes (total 300 samples), and for T2 only farrowing, dry, gilt and grower/finisher age classes were sampled (total 240 samples). For practicality, the samples collected at each time point per age class were pooled in the laboratory into six groups (10×1 g faeces per pool) for microbiological processing, so the numbers of pigs sampled per age class per time point could be increased. Samples of individual seagull faeces were also collected on farm, where possible for T1 (*n*=14) and T3 (*n*=15). Microbiological culture was performed as previously described [26], with indicator *E. coli* isolated using chromogenic medium CHROMagar ECC or ECC plates, ECC +ciprofloxacin (1 mg l^−1^) and ECC +cefotaxime (1 mg l^−1^). A maximum of 20 presumptive *E. coli *, appearing blue on chromogenic ECC plates, were purified per pooled sample and species confirmed subsequently via WGS analysis (see below). Variation in isolate numbers occurred due to presence or absence of isolates on antibiotic selective media and availability of pig age classes or seagull faeces for sampling. In total, 349 *E. coli* isolates from pigs and 36 *E. coli* isolates from seagull faeces were selected for further analysis.

### Minimum Inhibitory Concentration (MIC) determination

The MIC values for all isolates were determined by the Sensititre broth microdilution method (https://www.thermofisher.com/uk/en/home/clinical/clinical-microbiology/antimicrobial-susceptibility-testing.html). All 385 isolates were screened against a panel of antibiotics: ampicillin, azithromycin, cefotaxime, ceftazidime, chloramphenicol, ciprofloxacin, colistin, gentamycin, meropenem, nalidixic acid, sulfamethoxazole, tetracycline, tigecycline and trimethoprim (EUVSEC plates, Thermofisher).

### DNA extraction and short-read sequencing

A subset of 385 *E. coli* representative of the different pig age classes and seagull was selected for whole genome sequencing (WGS); 349 pig isolates and 36 seagull isolates were included. Genomic DNA was extracted and WGS performed on the isolates using the Illumina NextSeq platform, as described [9], with average coverage of 79-fold. *De novo* assembly of WGS reads was performed using SPAdes v3.12.0 [27], and the species identity for all 385 isolates was confirmed using the WGS reads with Kraken v1.0 utilising the MiniKraken DB_8 GB database [28]; they showed ≥90 % match to *E. coli *. Seven allele MLST was performed using ‘mlst’ v2.14 utilising the Warwick *E. coli* PubMLST scheme v1.1.2 [29]. The presence of acquired AMR genes was determined using the APHA SeqFinder V1 pipeline [30] which can be used to predict AMR phenotypes with ~98 % accuracy [31]; the criteria for determining gene presence using APHA SeqFinder was 100 % mapping of the query gene to the reference with no gaps or non-calls [32]. AMR arising from chromosomal gene mutations other than in *gyrA* or *parC* were not included. The *E. coli* serotypes were inferred *in silico* using SRST2 [33] which identifies the lipopolysaccharide (O) and flagellar (H) surface antigens types.

### Long-read sequencing

Fifteen isolates were selected for long-read sequencing based on AMR content. Total DNA was extracted from selected isolates for long-read sequencing using a QIAGEN Genomic-tip 100 G^−1^ kit. Individual isolate DNA was barcoded prior to sequencing with an Oxford Nanopore Rapid Barcoding Kit (SQK-RBK004).

Long-read sequencing was performed using the Oxford Nanopore MinION platform on a R9.4.1 flow cell. Barcodes were removed from reads using the Oxford Nanopore EPI2ME FASTQ BARCODING v3.10.2 workflow. Assembly of long-reads was performed using a hybrid assembly approach, with previously generated Illumina NextSeq short reads and Oxford Nanopore MinION long-reads, using Unicycler v0.4.7 [34]. MinION reads were mapped to completed Unicycler assemblies using the BWA-MEM algorithm of bwa v0.7.17-r1188 [35], and alignments visualised in Artemis v16.0.0 [36] to confirm coverage of regions of interest. Plasmid and transposon sequences were given preliminary annotation with Prokka, the sequences were further confirmed via the identification of all ORF’s over 100 amino acids in length, the ORF nucleotide sequences were then annotated with the highest scoring NCBI blast [37] hit. The annotated transposons sequences were visualised with EasyFig [38] and plasmids with BRIG [39]. Mob-typing was performed on the pIncY-T1M44 and pIncX-T1M17, for which resolved genomes were available using mob-typer [40].

### Phylogenetic and SNP analysis

Snippy version 4.3.6 [41] was used to detect SNPs in whole genomes of the 385 *E. coli* with respect to the *E. coli* K-12 MG1655 reference strain (GenBank accession number U00096.3). The full genome SNP alignment was used to generate a maximum-likelihood phylogenetic tree using RAxML Next Generation GTR+FO+G4 m model, which was rooted with the K-12 reference strain. A total of 4 641 652 alignment sites were used which comprised 3.02 % invariant sites, 10.32 % gaps and 455 859 patterns, 100 bootstrap replicates were performed. Final tree images and annotations were generated using iTOL v4 [42]. A full pairwise SNP distance matrix was generated (Table S1, available in the online version of this article) using snp-dists v0.6 (https://github.com/tseemann/snp-dists). Clones were identified from the SNP distance matrix, with the first isolate of the clonal group (by time point) used for determining SNP counts to assess clonality. A full pairwise SNP distance matrix was also generated following a full and core genome SNP alignment generated in Snippy using WGS of isolates within the outlier group and isolates from the same ST present in Enterobase (http://enterobase.warwick.ac.uk/) against the reference K-12 strain. The ‘Antibiotic Resistance Dynamics: the influence of geographic origin and management systems on resistance gene flows within humans, animals and the environment’ (ARDIG) study accession number from the European Nucleotide Archives is PRJEB39604 where assembled contigs are openly accessible. The raw reads for isolates from this dataset is also available in the NCBI nucleotide archive under project number PRJNA750276; the accession number of each isolate is included in Table S9.

## Results

### Diversity of *E. coli* isolates and their AMR

#### a) Phylogeny and MLST

**Fig. 1.**
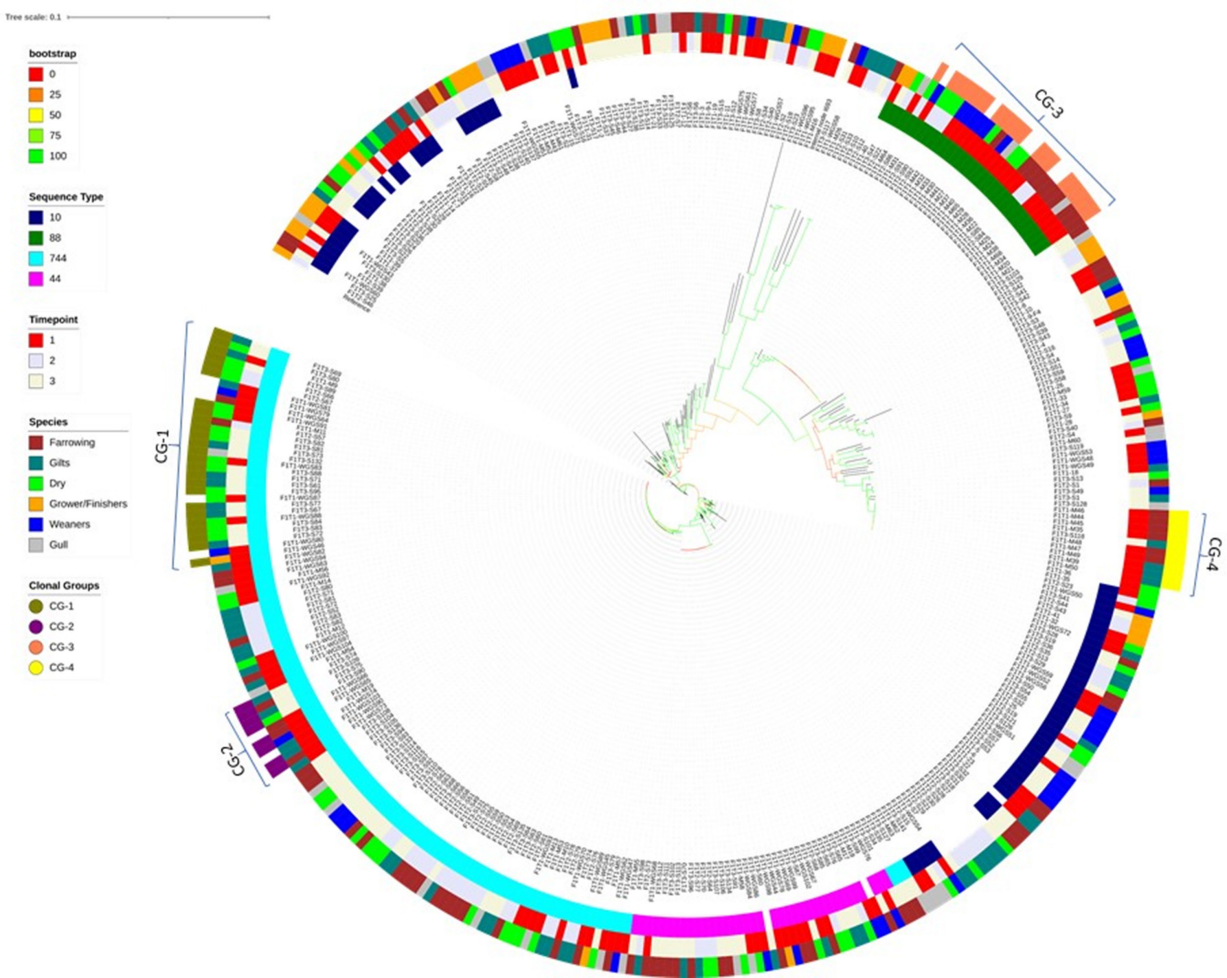
A whole genome SNP-based maximum-likelihood tree demonstrating phylogenetic relatedness of 385 *E. coli* isolates sequenced during the study. Genetic relatedness of isolates is demonstrated by branch length and confidence in clades by bootstrap confidence values, illustrated by coloured branches. Dominant STs (ring 1), time-points (ring 2) and source animal (ring 3) are illustrated by peripherally coloured rings. Clonal groups (CG) are demonstrated by brackets, while clones are highlighted in the outer ring (ring 4).

A total of 385 *E. coli* isolates were recovered from a farm with low antimicrobial usage (see Methods for details). The *E. coli* were recovered from pig faeces of five age classes (weaners: 46, gilts: 83, farrowing: 100, dry: 84, grower/finisher: 36; see Methods for description of each age class) and seagull [43] faeces over three time points: time point 1 (T1), time point 2 (T2), time point 3 (T3), separated by 6 months, on antibiotic selective and non-selective media (see Table S1). These isolates were analysed with short-read Whole Genome Sequencing (WGS) for their AMR genotype, Sequence Types (STs) and phylogeny. A bootstrapped SNP-based maximum-likelihood phylogenetic tree was constructed (Fig. S1) where the isolates mostly clustered in agreement with their ST, although some clusters were interspersed with isolates of unknown STs and an outlier group, comprising ten isolates from dry and farrowing sows, gilts and gulls, showed several thousand SNP differences to all other isolates (Fig S1, Table S1). The phylogenetic tree was redrawn excluding outliers to increase resolution of the remaining branches (Fig. 1). To confirm that the outliers were not due to any artefact, for seven isolates with known STs, a pairwise SNP distance matrix was generated with WGS from isolates of the same STs available from Enterobase (https://enterobase.warwick.ac.uk/). The results indicated these isolates were highly diverse to others from the same ST, except in those instances where two isolates may be from the same project (Table S2).

No large scale (>10 isolates) clustering was observed associated with pig age class or animal source with small and localised clustering observed by time point. Some ST clades comprised of particularly genetically homogenous populations (Table S1), such as ST744 (Fig. 1) containing 59 ST744 isolates which differed by a mean of 64 SNPs (±0.9 SEM). However, three ST744 isolates were less homogenous and clustered with other ST44s, indicating their core genome to be more similar to the latter type, which nevertheless belongs to the same clonal complex. This crossover also highlights the power of core genome phylogeny over the seven gene MLST in accurately identifying genetic relatedness. The ST44 and ST88 clades (Fig. 1) contained 36 ST44 and 27 ST88 isolates, differing by a mean of 40 (±0.84 SEM) and 18 (±0.49 SEM) SNPs, respectively, also indicating presence of closely related isolates. In contrast to other STs, ST10 showed much larger genetic differences with this clade containing 30 ST10 isolates that differed by an average of 6128 SNPs (±140.56 SEM; Table S1). Although ST10 has been associated with ExPEC pathotypes, it has also been reported from healthy pigs in the UK in multiple studies, so this is not an unusual finding [10, 12, 44].

Analysis of STs showed that diversity of the *E. coli* population isolated from non-selective media (ECC) was significantly higher (*P*=3.25E-28) than those isolated from ciprofloxacin (CIP) selective media (Shannon diversity indices H’ of 2.84 and 0.95 respectively). While 54 individual STs were identified from non-selective media, only seven STs were identified from CIP-containing media and eight from cefotaxime (CTX) media. These selective media populations were dominated by specific STs such as ST744 (CIP) and ST88 (CTX) (Table 1). A minimum spanning tree of MLST alleles demonstrated the majority of isolates from both non-selective and CIP-containing media belonged to the ST10 clonal complex (Fig. S2).

**Table 1.** Table of the four most frequently observed ST from each media across all time points, alongside count data for each ST and percentage proportion of total isolates for each media. Also shown are total isolate counts across all time points for each media (ECC: Chromagar ECC, CIP: Chromagar ECC+Ciprofloxacin, CTX: Chromagar ECC+Cefotaxime, ESBL: Chromagar ESBL) and associated Shannon Diversity Index *H’*. Unidentified or novel ST is indicated with ‘-’

ECC			CIP			CTX			ESBL		
ST	Count	Proportion (%)	ST	Count	Proportion (%)	ST	Count	Proportion (%)	ST	Count	Proportion (%)
10	64	35.4	744	107	68.6	88	27	73	58	8	66.7
–	29	16	44	36	23.1	2721	3	8.1	48	2	16.7
744	6	3.3	1421	3	1.9	58	2	5.4	4156	1	8.3
34	6	3.3	117	3	1.9	7401	1	2.7	10	1	8.3
**Total**	**181**		**Total**	**156**		**Total**	**37**		**Total**	**12**	
***H'***	2.84		***H'***	0.95		***H'***	1.08		***H'***	0.98	

#### b) AMR content

**Fig. 2.**
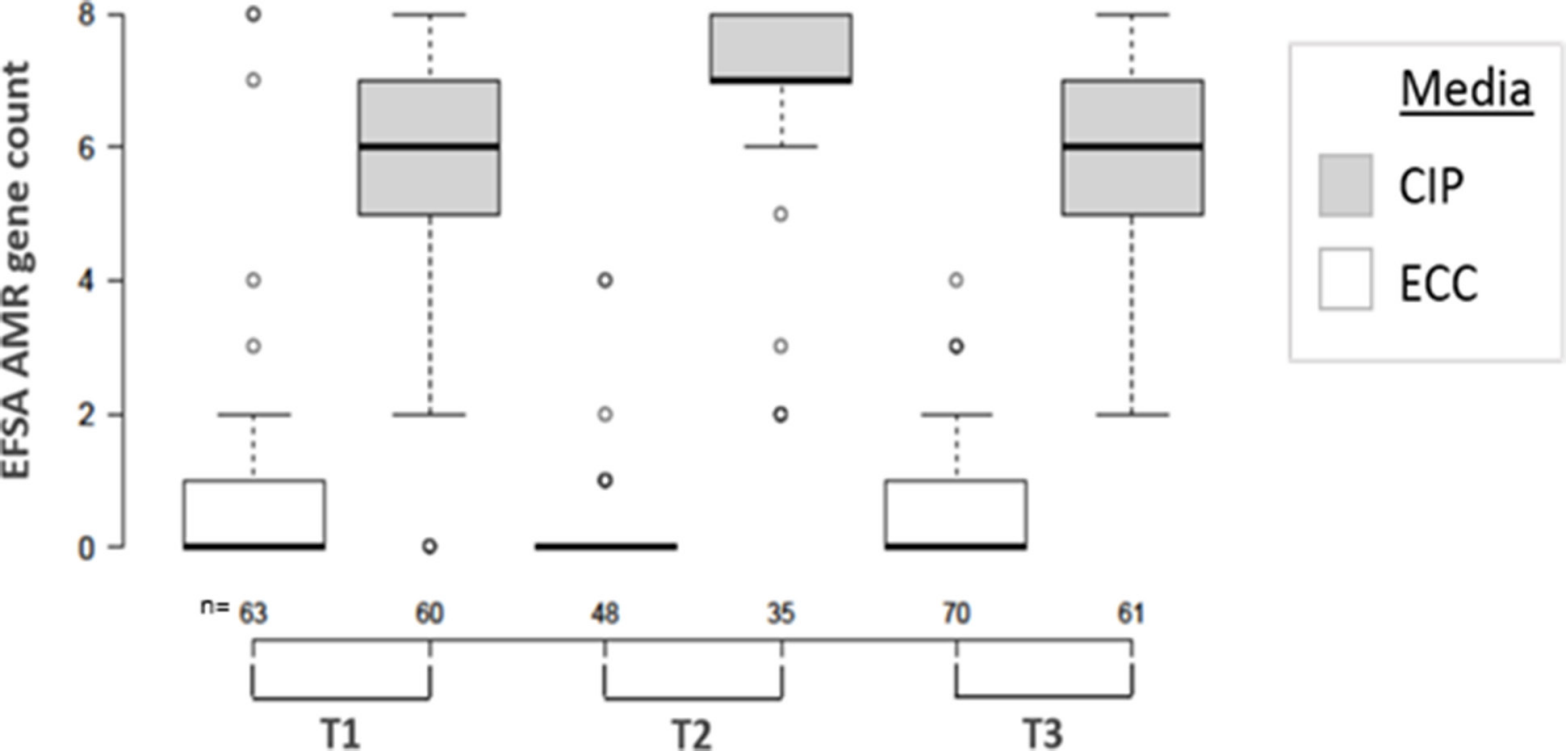
Comparison of AMR gene counts (including *gyrA* and *parC*) conferring resistance to antibiotics listed on the EFSA panel per isolate per time-point and isolation media. Isolation media is demonstrated by colour, and number of isolates analysed per category are indicated below on the X-axis.

Examination of the AMR gene content identified 41 AMR genes (Table S3). Among the 181 *E. coli* isolates from non-selective ECC media the total AMR genes was low with a mean count of 1.1 AMR genes per isolate, not including quinolone resistance-determining region (QRDR) mutations in *gyrA* and/or *parC*. Only seven isolates from non-selective ECC media (3.9%) were identified as genotypically Multi Drug Resistant (MDR; Fig. 2) i.e. harbouring resistance genes belonging to three or more antimicrobial classes [10]. The majority of isolates contained no resistance genes (63 % *n*=114), and the AMR gene content showed no statistically significant variation across all three time points (*P*=0.079). When only the genes conferring resistance to antimicrobials included in the European Food Safety Authority (EFSA) panel [45], which are of relevance to human therapeutics were considered, the proportion of isolates containing no relevant AMR genes increased to 71 % (*n*=128), with on average 0.6 AMR genes/isolate in isolates from non-selective ECC plates from all three time points (Fig. 2). When separated by age, class and source, the isolates from non-selective media demonstrated that the grower/finisher age class displayed the highest average AMR gene content (2.11 genes/isolate), followed by the weaner age class (1.33 genes/isolate). The age class with the lowest AMR gene content was the farrowing sows (0.46 genes/isolate).

For the 156 *E. coli* isolates from CIP-containing selective media, the AMR gene content was significantly higher compared to those from non-selective media for all time points (Fig. 2; Table S4). The mean count, for isolates recovered from CIP-containing selective media, was 8.9 AMR genes/isolate, across all time points, with 74 % (*n*=116) of these isolates identified as genotypically MDR. When only the genes conferring resistance to antimicrobials in the EFSA panel were considered, on average each isolate harboured resistance to six antimicrobials; which was significantly higher than isolates from non-selective media (*P*=1.12456E-90; Fig. 2). Of the 158 isolates identified with *gyrA* and/or *parC* QRDR mutations, only four (2.5 %) were isolated from non-selective media, with the majority (96 %, *n*=152) isolated from CIP-containing media.

The low isolate numbers from CTX-containing selective media (T1: *n*=26, T2: *n*=12, T3: *n*=3) prevented meaningful comparison of trends between time points of ESBL/AmpC bacteria, which were recovered from this plate [12]. However, the most frequently occurring β-lactamase identified in isolates from all time points, irrespective of the agar plate, was *bla*_TEM-1b_ (*n*=126), with 80 % (*n*=101) of *bla*_TEM-1b_ identified in ST744 isolates; other *bla*_TEM-1_ variants were identified in lower numbers (Table S3). Across all time points, 21 isolates were identified as containing *bla*_CTX-M_ genes with 52 % (*n*=11) harbouring *bla*_CTX-M-15_ (Table S3). Other isolates containing β-lactamases included two from T1 carrying *bla*_CARB-2_ and a T3 isolate from the farrowing pig age class (F1T3-S117) was identified as carrying both *bla*_CMY-2_ and *bla*_OXA-1_.

Plasmid associated fluoroquinolone resistance genes were observed in 11 isolates, bearing the *qnrS1* gene, which has previously been reported from pigs [46]; 10 of the 11 isolates belonged to ST58 where the *qnrS1* gene was associated with a IncY plasmid that also contained *bla*_CTX-M-15_ and a complete BREX phage resistance system (plasmid pIncY-T1M44, Fig. S3). In one seagull isolate (ST10), the *qnrS1* gene was associated with a plasmid containing the FIB replicon type but also contained *bla*_CTX-M-15_; this isolate additionally contained a *parC* mutation.

**Fig. 3.**
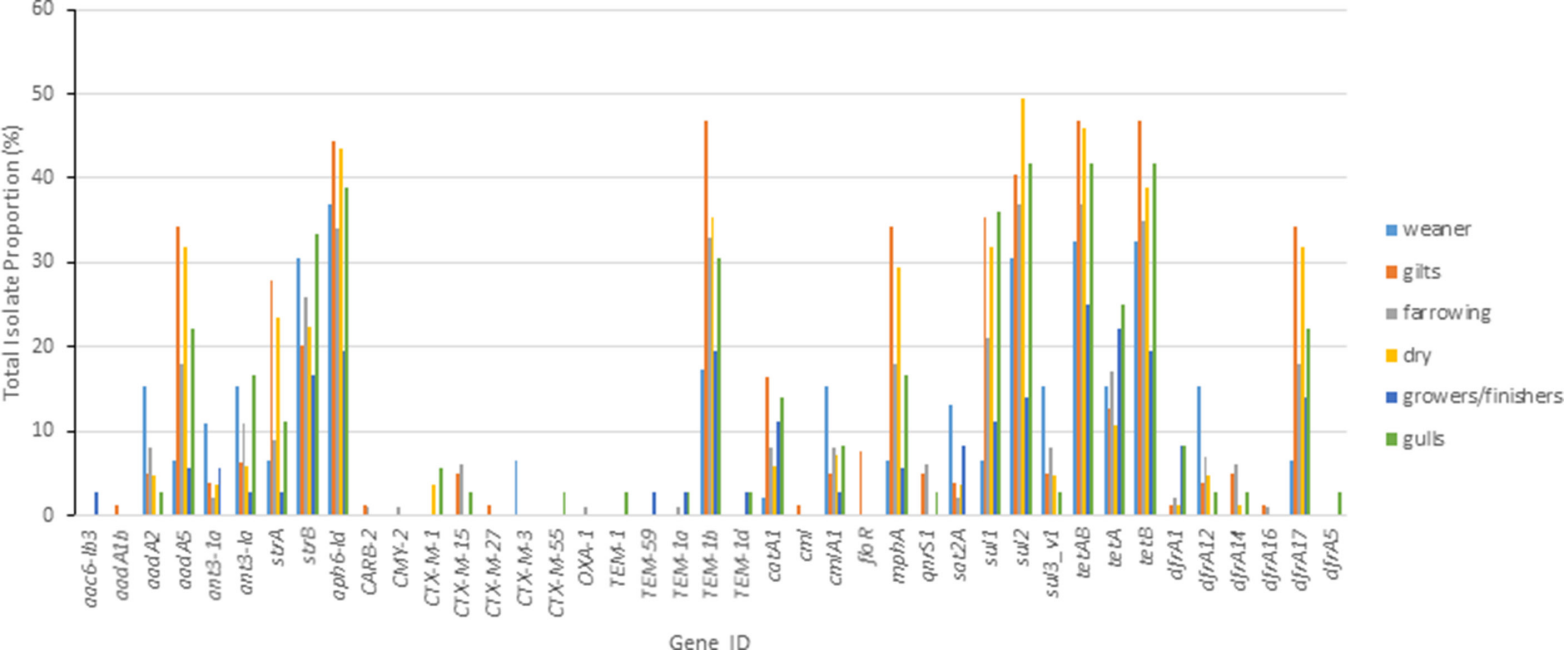
Proportion of total *E. coli* containing each AMR gene shown (see Table S2 for details). The isolates were collected from the duration of the study and all media (Weaner: *n*=46, Gilt: *n*=83, Farrowing: *n*=100, Dry: *n*=84, Grower/Finisher: *n*=36, Gull: *n*=36).

The proportion of isolates containing resistance genes, when isolates from all media (selective and non-selective) were combined, demonstrated that among pigs there were generally no particular AMR patterns for age class, with the exception of ESBL genes (Fig. 3). The farrowing and weaner age classes harboured the highest proportion of isolates containing ESBL genes. For gulls and pigs, the proportion of isolates containing AMR genes and the AMR classes they encoded were similar with exception of ESBL genes being a higher proportion in gulls.

There was a total of 63 unique AMR genotypes identified, with 116 isolates (30 %) harbouring no acquired AMR genes. Of the resistance genotypes given in Table 2, the ten most frequently observed accounted for 42 % (*n*=163) and were notably linked to *E. coli* STs. Six of the top ten most frequent resistance profiles were only associated with a single ST, three were associated only with ST744 and the remaining either with ST88, ST44 or ST58.

**Table 2.** AMR genotypes of 385 isolates as identified by APHA Seqfinder. Total count of isolates containing the genotype is shown alongside the ST most frequently associated with the genotype and fraction (in percentage) of isolates possessing the AMR genotype derived from this ST. Only genotypes with counts >3 isolates are shown

AMR Genotype	Count	ST (%)
*aadA5, aph3-Ia, strA, strB, bla*_TEM-1b_ *, int1, mphA, sul1, sul2, tetAB, dfrA17, gyrA, parC*	28	744 (100)
*aadA5, strA, strB, bla*_TEM-1b_ *, int1, mphA, sul1, sul2, tetAB, dfrA17, gyrA, parC*	28	744 (100)
*aph3-Ia, strA, strB, sul2, tetAB*	20	88 (100)
*gyrA, parC*	20	44 (90)
*aadA2, ant3-Ia, cmlA1, int1, sul3_v1, tetA, dfrA12, gyrA, parC*	16	44 (100)
*tetA(B*)	13	10 (35)
*aadA5, strA, strB, bla*_TEM-1b_ *, catA1, int1, mphA, sul1, sul2, tetA(B), dfrA17, gyrA, parC*	11	744 (100)
*tetA*	10	10 (50)
*bla*_CTX-M-15_ *, bla*_TEM-1b_ *, qnrS1, tet(A), dfrA14*	9	58 (100)
*ant3-1a, sat2A*	8	10 (75)
*aph3-Ia, strA, strB, bla*_TEM-1b_ *, sul2, tetAB, gyrA, parC*	7	744 (100)
*aadA5, aph3-Ia, strA, strB, bla*_TEM-1b_ *, catA1, int1, mphA, sul1, sul2, tet(A)B, tetA, dfrA17, floR, gyrA, parC*	7	744 (100)
*sul2*	6	88 (100)
*strA, strB, tetA(B*)	5	10 (40)
*aadA5, aph3-Ia, strA, strB, bla*_TEM-1b_ *, catA1, int1, mphA, sul1, sul2, tetAB, dfrA17, gyrA, parC*	5	744 (100)
*aadA2, ant3-Ia, cmlA1, int1, sul3_v1, dfrA12, gyrA, parC*	5	44 (80)
*aph3-Ia, strA, strB, bla*_TEM-1b_ *, catA1, int1, sul2, tetA(B), gyrA, parC*	4	744 (100)
*strA, strB, bla*_TEM-1b_ *, catA1, int1, sul2, tetAB, dfrA17, gyrA, parC*	3	117 (100)
*strA, strB, bla*_CTX-M-3_ *, tetA(B*)	3	48 (100)
*bla*_CTX-M-1_ *, sul2, tetA*	3	2721 (100)
*ant3-Ia, int1, sul1, tetA, dfrA1*	3	744 (100)
*strA, strB, bla*_TEM-1b_ *, sul2, tetA(B), gyrA, parC*	3	744 (100)
*aph3-Ia, strB, bla*_TEM-1b_ *, sul2, tetA(B), gyrA, parC*	3	744 (100)

Phenotypic testing (MIC measurement) was performed for all 385 *E. coli* isolates included in the study against a panel of 14 antimicrobial to see whether the isolates expressed their genotypic resistances (Table 3). The results showed that for all antimicrobial classes in which AMR genes had been detected (Table S3), isolates expressed the corresponding phenotypic resistance e.g. beta-lactamases (ampicillin, ceftazadine and cefoxatin), or fluoroquinolone (ciprofloxacin, naladixic acid); whilst those antimicrobial families with no AMR genes detected in isolates also showed no corresponding resistance phenotype e.g. colistin, meropenem. This suggested that although exposure of pigs and gulls to on-farm antimicrobials may be different as pigs were permanently present, but gulls were transitory, it did not influence expression of AMR genes in *E. coli* isolated from the different compartments (Table 3).

**Table 3.** Antimicrobial susceptibility values for all *E. coli* isolates. The MICs were determined by broth microdilution and interpreted using ECOFF cut-off values (in columns) in μg ml^−1^ and show the numbers of isolates (in rows) with MIC above these values for each antimicrobial tested. Full description of the each antimicrobial is given in Methods

	AMP	AZI	FOT	TAZ	CHL	CIP	COL	GEN	MERO	NAL	SMX	TET	TGC	TMP
ECOFFs:	**>8**	**>16**	**>0.25**	**>0.5**	**>16**	**>0.064**	**>2**	**>2**	**>0.125**	**>16**	**>64**	**>8**	**>1**	**>2**
all: *n*/385	**175**	**90**	**48**	**45**	**85**	**170**	**0**	**0**	**0**	**161**	**193**	**226**	**0**	**150**
%	**45%**	**23%**	**12%**	**12%**	**22%**	**44%**	**0%**	**0%**	**0%**	**42%**	**50%**	**59%**	**0%**	**39%**
pig: *n*/349	**158**	**83**	**42**	**39**	**77**	**156**	**0**	**0**	**0**	**147**	**172**	**201**	**0**	**135**
%	**45%**	**24%**	**12%**	**11%**	**22%**	**45%**	**0%**	**0%**	**0%**	**42%**	**49%**	**57%**	**0%**	**39%**
gull: *n*/36	**17**	**7**	**6**	**6**	**8**	**14**	**0**	**0**	**0**	**14**	**21**	**25**	**0**	**15**
%	**47%**	**19%**	**17%**	**17%**	**22%**	**39%**	**0%**	**0%**	**0%**	**39%**	**58%**	**69%**	**0%**	**42%**

*In silico* serotyping was performed and showed the panel of *E. coli* to harbour a plethora of O and H antigens. A number of isolates (Table S5) harboured O-antigens commonly reported from human enteric pathogens (e.g. O8, O15, O16, O86, O91, O98, O113, O128, O149, O164, O179 and O180 [47, 48]. In future, typing the virulence genes that can be present within enteric *Escherichia* sp., including *E. coli* [49–51] from livestock, will provide better understanding of the potential, if any, of these isolates to cause disease in humans if they pass through the food chain.

### Vertical transmission and clonal transfer affects AMR transmission and persistence

Bacterial clones were defined by comparing SNPs identified from genomic alignment to *E. coli* K-12 substrain MG1655 (4.641 Mb) and by using estimates of natural *E. coli* mutation rates between 2.26×10^−7^ and 3.0×10^−6^ substitutions per bp per year [52, 53]. Therefore, 1–14 SNPs would be expected in an *E. coli* clone over the duration of 12 months, and isolates differing by ≤14 SNPs, using a pairwise SNP distance matrix, were defined as clones (Table S1 and S6). Multiple, large clonal groups (CG-1 to CG-4, Fig. 1) were identified that comprised *E. coli* isolates conforming to this definition. The bootstrap confidence value of clades harbouring the clonal groups was >99 %, providing high confidence in these branches.

Whole genome SNP analysis identified the CG-1 clonal group (Fig. 1), which accounted for 22 % of the total ST744 isolates; it included 25 isolates identified as clonal with respect to the first isolate F1T1-M11 (Table 4). They were present across all time points (T1 : 5 isolates, T2 : 3 isolates, T3 : 16 isolates) and in multiple pig age classes and a gull sample. The AMR genotype of the CG-1 clonal group is detailed in the next section.

**Table 4.** ST 744 isolates belonging to the CG-1 clonal group (reference isolate F1T1-M11) alongside SNP differences in comparison to reference isolate, isolate source and AMR genotype

Source	Isolate ID	SNPs	AMR Gene Pattern
Dry	F1T1-M11	n/a	*aadA5, aph3-Ia, strA, strB, bla*_TEM-1b_ *, int1, mphA, sul1, sul2, tetA(B), dfrA17, gyrA, parC*
Gilts	F1T1-M9	2	*aadA5, strA, strB, bla*_TEM-1b_ *, int1, mphA, sul1, sul2, tetA(B), dfrA17, gyrA, parC*
Growers	F1T1-WGS46	3	*aadA5, aph3-Ia, strA, strB, bla*_TEM-1b_, *int1, mphA, sul1, sul2, tetAB, dfrA17, gyrA, parC*
Dry	F1T1-WGS83	8	
Dry	F1T1-WGS88	4	
Dry	F1T1-WGS91	4	
Farrowing	F1T2-S57	3	
Dry	F1T2-S66	1	
Dry	F1T2-S67	2	
Gull	F1T3-S132	9	
Gilts	F1T3-S61	3	
Gilts	F1T3-S67	3	
Gilts	F1T3-S69	4	
Gilts	F1T3-S71	4	*aadA5, aph3-Ia, int1, mphA, sul1, tetAB, dfrA17, gyrA, parC*
Gilts	F1T3-S72	5	*aadA5, aph3-Ia, strA, strB, bla*_TEM-1b_, *int1, mphA, sul1, sul2, tetAB, dfrA17, gyrA, parC*
Gilts	F1T3-S73	6	
Gilts	F1T3-S77	5	
Dry	F1T3-S80	5	
Dry	F1T3-S81	7	
Dry	F1T3-S82	6	
Dry	F1T3-S83	4	
Dry	F1T3-S84	7	
Dry	F1T3-S88	8	
Dry	F1T3-S89	7	
Dry	F1T3-S95	5	

A second clonal group CG-2 (Fig. 1) comprised eight ST744 isolates, where F1T1-M19 was identified as the first incidence of the clone. Clones were identified as having persisted between T1 and T3, with four identified in T1 and a further four in T3. The majority of isolates (63 %, *n*=5) within CG-2 possessed the AMR genotype *aph3-Ia*, *strA*, *strB*, *bla*_TEM-1b_, *sul2* and *tetAB* although three T3 isolates lacked *strA*.

Clonal dissemination and persistence was not limited to ST744 isolates but also identified in ST88, where clonal group CG-3 (Fig. 1) comprised 21 isolates identified as clonal to the primary isolate F1T1-M27 that persisted between T1 and T2 from the farrowing, weaners and dry age classes, alongside two T1 gull isolates. The majority of isolates (81 %; *n*=17) possessed the AMR genotype *aph3-Ia*, *strA*, *strB*, *sul2* and *tetAB*, which was associated with an IncF replicon type and was the third most frequent genotype observed (Table 2).

Clonal group CG-4 included ten isolates of ST58 from only the gilt and farrowing age classes identified as clonal to the primary isolate F1T1-M35. This clone was identified as having persisted between T1 and T3, with all isolates except F1T3-S118 having been identified from T1. All ten CG-4 isolates harboured the IncY plasmid pIncY-T1M44 (Fig. S3), which was exclusively associated with this clonal group. pIncY-T1M44 did not contain the molecular machinery associated with conjugative or mobilizing plasmids, such as transfer (*tra*) genes or a mobilization (*mob*) genes (Table S7 and S8; [40, 43, 54].

A broader group was identified within the ST44 *E. coli* population. A single T1 ST44 gull isolate (F1T1-M58) was identified as clonal to two T2 isolates and two T3 isolates from pig sources (12–14 SNPs apart), as well as being closely related (15–28 SNPs) to a further 18 T2 and T3 isolates from pigs (weaner, farrowing, dry and gilt age classes) and a single T3 gull isolate. Within the ST44 group, 15 isolates, including the gull isolate F1T1-M58, harboured a 39, 585 bp non-conjugative IncX1 plasmid (pIncX-T1M17, Fig. S3). Similar to pIncY-T1M44, pIncX-T1M17 was missing *tra* and *mob* genes (Table S7 and S8); it harboured the resistance genes *aadA2*, *ant3-Ia*, *cmlA1*, *int1*, *sul3_v1*, *tetA* and *dfrA12* (Fig S3). Three T3 (13%), harboured a truncated version of the pIncX-T1M17 plasmid (34,426 bp) which was missing the *tet* resistance operon. Both the complete and truncated pIncX-T1M17 plasmids were exclusively associated with ST44 isolates.

Dissemination of *E. coli* clones between gulls and pigs was relatively frequent with 31 % (*n*=11) of the total isolates from gulls in T1 and T3 having clones identified in pigs from ST44, 88 and 744 (Table 3). The clones identified from gull samples were also identified from multiple time points in pig samples, indicating persistence and recycling between the groups.

### Chromosomal integration enables AMR gene persistence in clonal ST744 populations

**Fig. 4.**
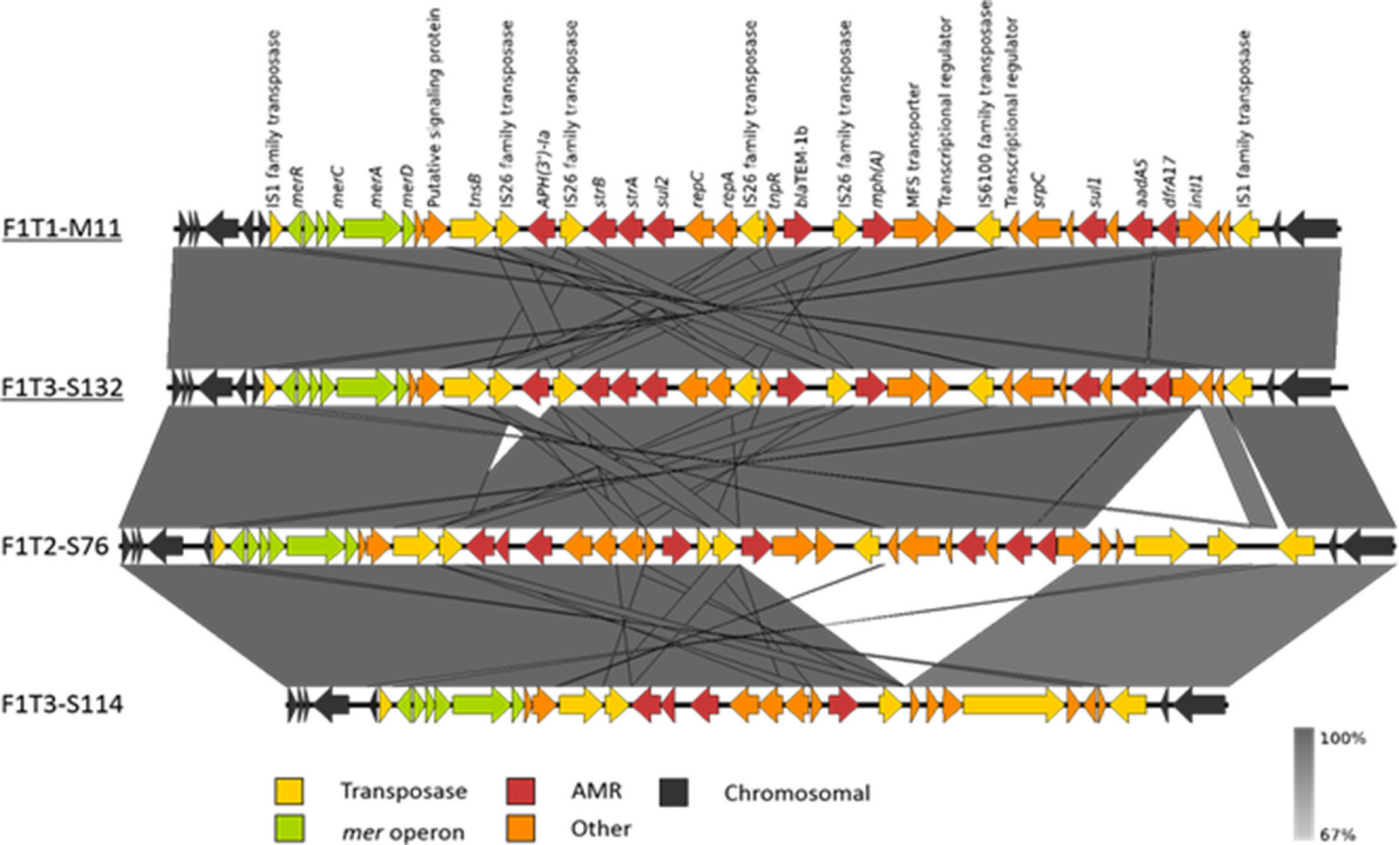
Comparison of the IS1 transposon region of four ST744 isolates (F1T1-M11, F1T3-S132, F1T2-S76 and F1T3-S114), confirmed by hybrid assembly, including two isolates from CG-1 clone (underlined). Nucleotide sequence identity range is demonstrated by gradient scale and gene types and orientations are labelled with coloured arrows; gene annotations are shown above F1T1-M11.

A near closed genome of the ST744 isolate F1T1-M11 with good long- and short-read coverage was used to accurately elucidate the mobile genetic element(s) harbouring the AMR genes. The AMR genes were integrated within the ST744 chromosome in a 27.7 kb transposon, flanked by IS1 family transposases and containing multiple IS*26* transposable units within. The 27.7 kb transposon contained an AMR gene cassette with *aadA5*, *aph3-Ia*, *strA*, *strB*, *bla*_TEM-1b_, *mphA*, *sul1*, *sul2*, *tetAB*, *dfrA17* and *intI1* genes, as well as an IncQ1 replicon and the complete mercury resistance operon (Fig. 4). The AMR gene cassette identified in F1T1-M11 was identified as being conserved in 22 further CG-1 clones (Table 4) and the associated AMR genotype was jointly the most frequently observed AMR genotype (Table 2). Five closely related isolates, with between 17–24 SNP differences to CG-1 clone F1T1-M11, harbouring this resistance pattern were included in the CG-1 group (F1T1-WGS64, F1T1-WGS79, F1T1-WGS80, F1T1-WGS81, F1T1-WGS87) due to their proximity on the phylogenetic tree (Fig. 1). The complete transposon was also identified by long/short-read hybrid assembly as being fully conserved in a T3 seagull CG-1 clone F1T3-S132 (Fig. 4) indicating persistence and possible transmission between pigs and wild birds during the sampling time.

Analysis of the resolved genomes from two ST744 isolates outside of CG-1 (F1T2-S76 and F1T3-S114; Fig. 4), but with similar AMR profiles (*aadA5*, *strA*, *strB*, *bla*_TEM-1b_, *catA1*, *intI1*, *mphA*, *sul1*, *sul2*, *tetAB*, *dfrA17* and *aadA2*, *ant3-Ia*, *strA*, *strB*, *bla*_TEM-1b_, *cmlA1*, *intI1*, *sul2*, *sul3_v1*, *dfrA12* respectively) demonstrated that although the IS*1* transposon region varied in length due to variation in AMR gene content, the IncQ1 replicon and mercury resistance operon remained conserved. Analysis of these ST744 isolates indicated that the IS*1* transposon had inserted in the same chromosomal location between *icsA* and *ymgF* in all (Fig. 4). Chromosomal integration of AMR genes was also identified in selected isolates from other STs such as ST963 and ST48 with resolved genomes (Accession: PRJEB39604).

## Discussion

It is well known that food animals may be a reservoir of AMR that affect humans [55–57]. In this study, we investigated an outdoor pig farm with low antimicrobial usage, in comparison to conventional pig farms in the Great Britain [10], for its AMR burden using detailed genomic analysis. Overall, the proportion of commensal *E. coli* isolates harbouring any resistance gene or chromosomal mutation resulting in resistance was low (~37 %), as determined from commensal *E. coli* recovered from non-selective media, with only 7 % being MDR; the level was even lower when only the EFSA panel of antimicrobials were considered (29%). Antimicrobial susceptibility testing to a panel of 14 antimicrobials indicated that isolates harbouring AMR genes expressed these resistances, despite being collected from farm environments with low AMU, and from birds that may only be present transitorily. These genotypic AMR levels were significantly lower than from conventional pig farms as evidenced in a recent cross-sectional study which has shown 70.9 % of *E. coli* isolated on non-selective plates from pigs on 56 UK farms, harboured any AMR gene or chromosomal mutation resulting in resistance; with 35 % being genotypically MDR [10], which is similar to the average AMR reported across Europe in 2017 [58]. In this study however, the AMR gene content was significantly higher in isolates from CIP-containing media, with up to 74 % being genotypically MDR, indicating presence at levels similar to previous reports from UK pig farms with conventional antimicrobial usage [26, 59]. The resistance gene contents of *E. coli* isolates nevertheless remained relatively constant over the 12 month period of sample collection, with persistence of AMR genes within specific lineages or clones of *E. coli* from both pigs and gulls. Also, the presence of AMR genes within non-conjugative plasmids, evidenced by the lack of conjugative or mobilizing genes [43, 54] within isolate genomes, suggested vertical rather than horizontal expansion of AMR elements.

De Been *et al.* [53] have previously noted presence of epidemiological linkage between isolates from farmers and their pigs, indicating transmission between these groups. In this study several clonal groups/lineages were identified within STs 88, 44 and 58 which were <14 SNPs apart indicating epidemiological linkage and persistence between multiple time points. Identification of ST744 clones from pigs, which persisted over the study duration, and its presence in some seagull samples, may also suggest wider transmission and recycling, as gulls may be exposed to isolates from anthropogenic sources due to their scavenging activities [21, 22]. In fact ST744 have been detected in wild bird populations in Germany [60] containing similar AMR profiles reported here, as well as cattle in France, but also containing ESBL genes, where they are documented to have spread clonally over significant distances [61]. Although our findings indicate that the same MDR *E. coli* clones occurred both in pigs and seagulls, the frequency and direction of transfer of these clones between these species, or even within different pig age classes (e.g. farrowing sows and weaners), and the degree of persistence in each species or pig age class were not established. This was due to a much smaller number of gull samples being available, as well as due to the difficulty of tracking individual weaners, sows and grower/finishers lineages on the pig farm over the time period sampled. Further work is required for a full understanding of transmission both between pig classes and the different animal species.

In addition, our findings of widespread chromosomal integration of AMR genes and likely proliferation of clones contrasts with other studies where transfer and persistence of AMR plasmids in a more heterogeneous population of *E. coli* on livestock farm, including pig farms, is common [9, 26, 59, 62]. Our study design may partially select for isolates with chromosomally encoded resistance genes, as the use of ciprofloxacin-containing media will select those with *gyrA* and *parC* mutations. However, this would not account for the high levels of chromosomally integrated resistance genes for antimicrobials of other classes such as those found in ST744 isolates, nor the apparent lack of conjugable plasmids identified. The persistence of MDR isolates in the absence of antimicrobials is likely to have been aided by the co-localisation of AMR genes with other genes possibly conferring increased fitness, such as mercury resistance within the ~27 Kb transposon of ST744 isolates or the BREX phage resistance system of the plasmid pIncY-T1M44 identified in ST58s. The co-localisation and co-occurrence of AMR genes with those associated with resistance to heavy metals has been extensively documented in literature, as well as the presence of heavy metals such as copper and zinc within pig manure [63–65]; although heavy metal usage on this farm was not known we can speculate that there is a link which requires further investigation.

In conclusion, our work suggests that reduction in antimicrobial usage on an outdoor pig farm likely reduces levels of *E. coli* isolates possessing AMR genes, and their possible transmission through the food chain. However, a basal MDR *E. coli* population persisted throughout the time period of this study, potentially due to transmission and recycling of clones between pigs of different age class, as well as wild birds that were present on this outdoor pig farm. This is in contrast to other studies which mostly report AMR gene transmission and persistence through horizontal gene transfer. Other co-selectors such as heavy metal contaminants may also have contributed to persistence of MDR *E. coli* isolates. AMR is a recognised global threat, and our study highlights how multiple farm factors, including the environment, should be considered in addition to antimicrobial usage to help mitigate the risk posed by rising resistances. In addition, on low AMU farms caution must been taken in any re-introduction of AMU to conventional levels, as this would likely reverse the low levels and the overall AMR burden.

## Supplementary Data

Supplementary material 1

Supplementary material 2
