# Supplementary material for: Use of genomics to explore AMR persistence in an outdoor pig farm with low antimicrobial usage
Source: Microb Genom. 2022 Mar 28;8(3):000782. doi: 10.1099/mgen.0.000782 (PMC9176276; doi:10.1099/mgen.0.000782)
Supplement: Supplementary material 1 [file mgen-8-0782-s001.pdf]

Use of genomics to explore AMR persistence in an outdoor pig farm with low antimicrobial usage.

Nathaniel Storey, Shaun Cawthraw, Olivia Turner, Margherita Rambaldi, Fabrizio Lemma, Robert Horton, Luke Randall, Nicholas A. Duggett, Manal AbuOun, Francesca Martelli, Muna F. Anjum.

Supplementary Figures and Tables

Figure S1. A SNP-based maximum-likelihood tree demonstrating phylogenetic relatedness of 385 *E. coli* isolates sequenced during the study, with branch lengths shown. The branch length indicates genetic relatedness and has been used to define the outlier group, labelled A. The outlier group are further highlighted in Supplementary Table S1 on pairwise SNP distances between isolates.

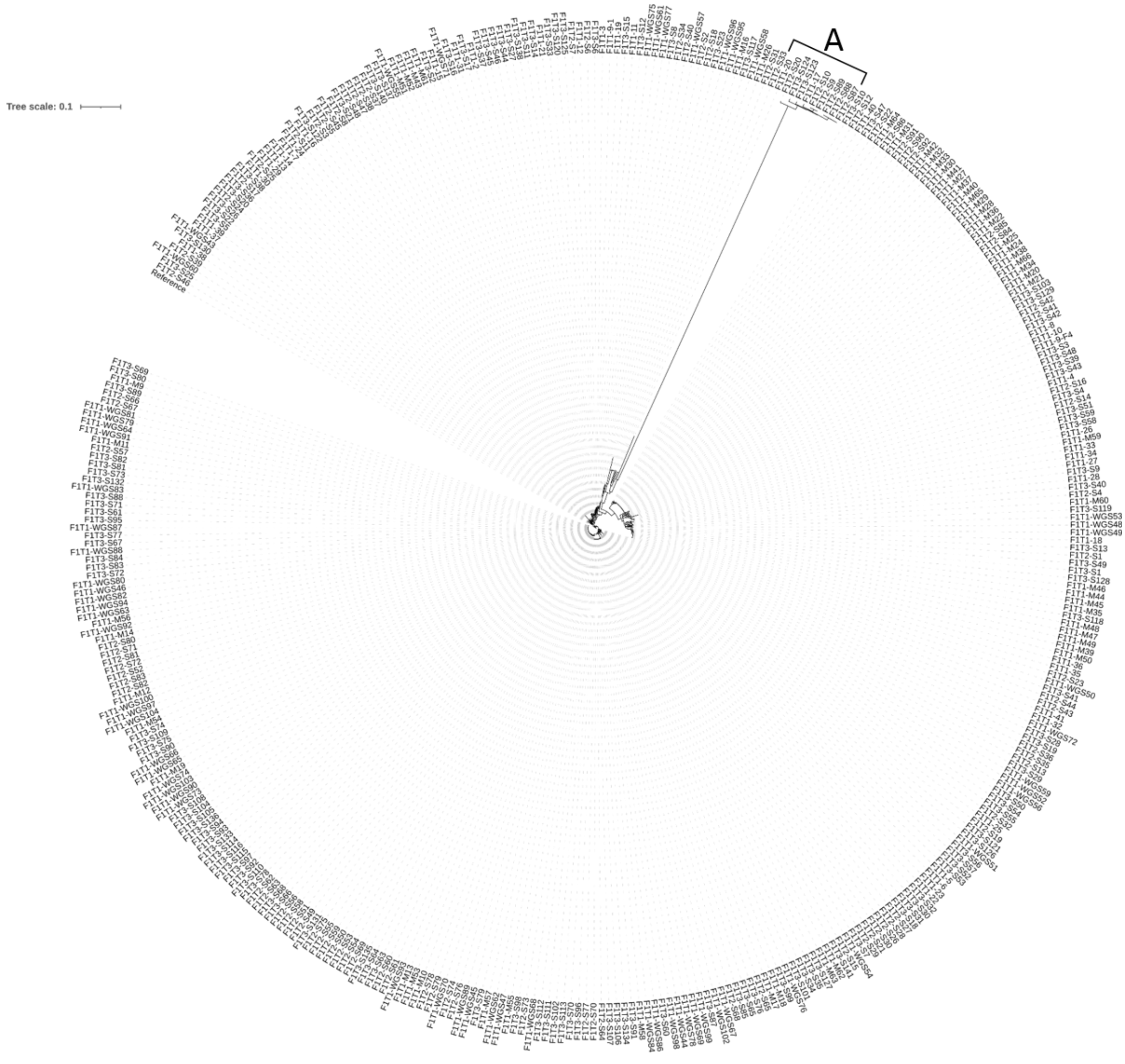

Fig S2: Minimum spanning tree demonstrating 62 STs of 346 *E. coli* isolates with selection media. Coloured pie charts denote isolation media distribution of ST, circle size is proportional to number of isolates, predicted clonal complexes denoted in grey. Dotted lines indicate connections between STs with differences of >3 loci. Isolates with no identified ST were excluded from the analysis.

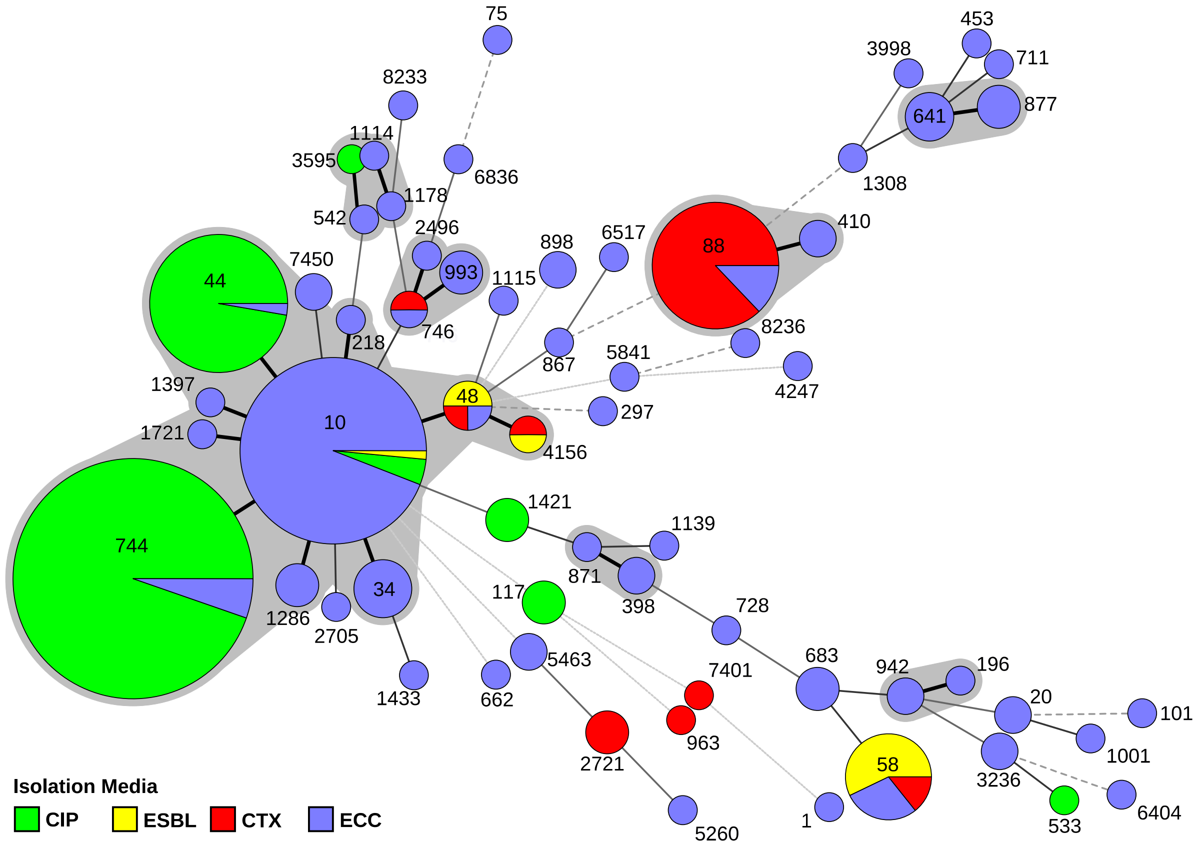

Figure S3. Plasmid maps of two plasmids described in this study, pIncY-T1M44 and pIncX-T1M17.Antibiotic resistance genes are indicated in red and BREX phage-resistance genes in blue. Isolates harbouring <u>pIncX-T1M17</u> are: F1T3-S96, F1T3-S99, F1T2-S65, F1T2-S68, F1T1-M58, F1T1-WGS86, F1T1-WGS98, F1T1-WGS69, F1T1-WGS78, F1T1-WGS99, F1T1-WGS67, F1T1-WGS102, F1T1-WGS76, F1T1-M17, F1T1-M18; and isolates harbouring <u>pIncY-T1M44</u> are: F1T1-M46, F1T1-M44, F1T1-M35, F1T1-M45, F1T1-M48, F1T1-M47, F1T1-M49, F1T1-M50, F1T1-M39, F1T3-S118.

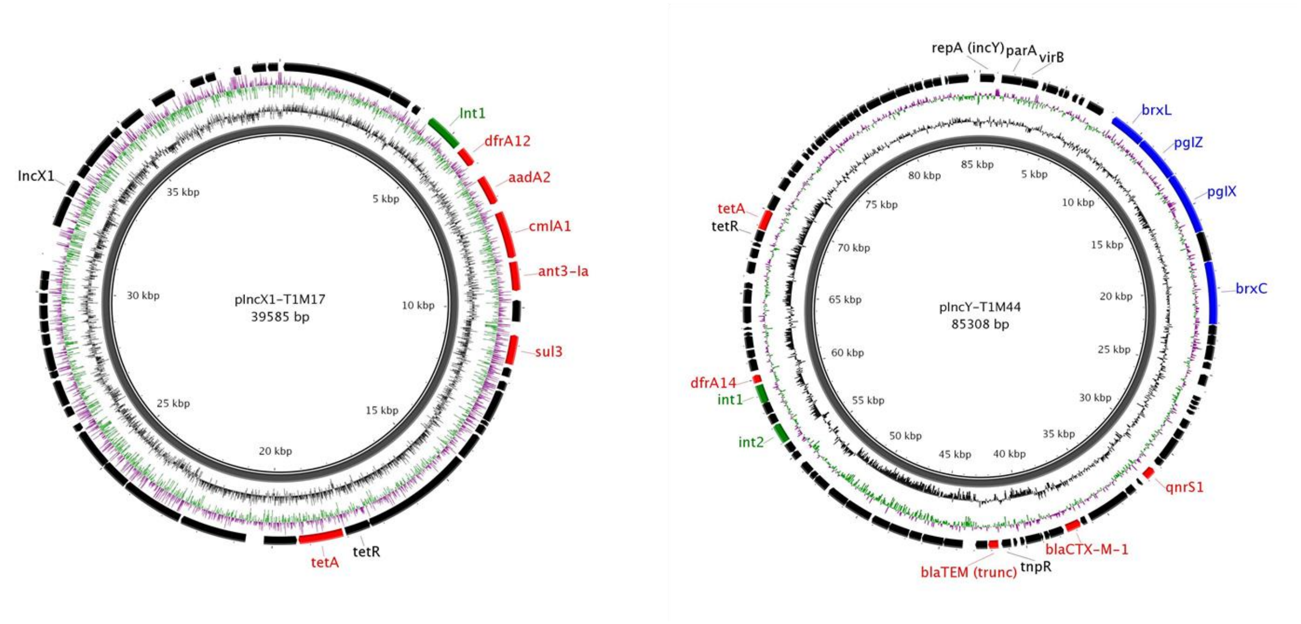

FA

Table S3. AMR gene counts by source and time-point as identified by the APHA SeqFinder pipeline, total counts are indicated in grey. *-indicates presence of mutations in the *ampC* promoter region associated with resistance; **- indicates presence of mutation in the QRDR regions of *gyrA* and *parC* associated with fluoroquinolone resistance.

	Dry			Dry Total	Farrowing			Farrowing Total	Gilts			Gilts Total	Grower/Finisher			Grower/ Finishers Total	Gull		Gull Total	Weaners		Weaners Total	Grand Total
	**T1**	**T2**	**T3**		**T1**	**T2**	**T3**		**T1**	**T2**	**T3**		**T1**	**T2**	**T3**		**T1**	**T3**		**T1**	**T3**		
**amino**																							
aac6-Ib3	0	0	0	0	0	0	0	0	0	0	0	0	0	1	0	1	0	0	0	0	0	0	1
aadA1b	0	0	0	0	0	0	0	0	0	0	1	1	0	0	0	0	0	0	0	0	0	0	1
aadA2	0	2	2	4	5	0	3	8	2	0	2	4	1	0	0	1	1	0	1	5	2	7	25
aadA5	9	6	12	27	4	10	4	18	5	11	11	27	2	0	0	2	4	4	8	3	0	3	85
ant3-1a	2	0	1	3	1	0	1	2	4	1	0	5	1	0	1	2	0	0	0	2	3	5	17
ant3-Ia	0	3	2	5	5	0	6	11	2	0	3	5	2	0	0	2	2	4	6	5	2	7	36
aph3-Ia	9	2	9	20	6	2	1	9	7	6	9	22	1	0	0	1	3	1	4	3	0	3	59
strA	12	11	12	35	17	11	6	34	11	13	10	34	6	0	2	8	8	7	15	14	3	17	143
strB	12	11	14	37	17	11	6	34	10	13	12	35	5	0	2	7	7	7	14	14	3	17	144
**betaL**																							
*bla*CARB-2	0	0	0	0	1	0	0	1	1	0	0	1	0	0	0	0	0	0	0	0	0	0	2
*bla*CMY-2	0	0	0	0	0	0	1	1	0	0	0	0	0	0	0	0	0	0	0	0	0	0	1
*bla*CTX-M-1	0	3	0	3	0	0	0	0	0	0	0	0	0	0	0	0	0	2	2	0	0	0	5
*bla*CTX-M-15	0	0	0	0	6	0	0	6	3	0	1	4	0	0	0	0	0	1	1	0	0	0	11

*bla*CTX-M-27	0	0	0	0	0	0	0	0	1	0	0	1	0	0	0	0	0	0	0	0	0	0	1
*bla*CTX-M-3	0	0	0	0	0	0	0	0	0	0	0	0	0	0	0	0	0	0	0	3	0	3	3
*bla*CTX-M-55	0	0	0	0	0	0	0	0	0	0	0	0	0	0	0	0	0	1	1	0	0	0	1
*bla*OXA-1	0	0	0	0	0	0	1	1	0	0	0	0	0	0	0	0	0	0	0	0	0	0	1
*bla*TEM-1	0	0	0	0	0	0	0	0	0	0	0	0	0	0	0	0	1	0	1	0	0	0	1
*bla*TEM-59	0	0	0	0	0	0	0	0	0	0	0	0	0	1	0	1	0	0	0	0	0	0	1
*bla*TEM-1a	0	0	0	0	1	0	0	1	0	0	0	0	0	0	1	1	1	0	1	0	0	0	3
*bla*TEM-1b	9	7	14	30	15	11	7	33	11	11	15	37	6	1	0	7	3	8	11	4	4	8	126
*bla*TEM-1d	0	0	0	0	0	0	0	0	0	0	0	0	1	0	0	1	0	1	1	0	0	0	2
**ampC*	3	4	0	7	9	2	0	11	0	0	0	0	0	0	0	0	2	0	2	7	0	7	27
**chlor**																							
catA1	4	0	1	5	6	1	1	8	3	10	0	13	4	0	0	4	5	0	5	1	0	1	36
cml	0	0	0	0	0	0	0	0	1	0	0	1	0	0	0	0	0	0	0	0	0	0	1
cmlA1	2	2	2	6	5	0	3	8	2	0	2	4	1	0	1	2	1	2	3	5	2	7	30
floR	0	0	0	0	0	1	0	1	0	6	0	6	0	0	0	0	0	0	0	0	0	0	7
**macro**																							
mphA	7	6	12	25	4	10	4	18	5	11	11	27	2	0	0	2	2	4	6	3	0	3	81
**quino**																							
qnrS1	0	0	0	0	6	0	0	6	3	0	1	4	0	0	0	0	0	1	1	0	0	0	11
***gyrA*	12	10	18	40	16	12	17	45	11	13	17	41	6	0	0	6	6	7	13	9	3	12	157
***parC*	12	10	18	40	16	12	17	45	10	13	17	40	6	0	0	6	6	7	13	9	3	12	156

**strep**																							
sat2A	2	0	1	3	1	0	1	2	3	0	1	4	0	2	1	3	0	0	0	3	3	6	18
**sulph**																							
sul1	8	7	12	27	4	10	7	21	5	11	12	28	3	1	0	4	5	8	13	3	0	3	96
sul2	14	14	14	42	18	13	6	37	9	11	12	32	5	0	0	5	8	7	15	11	3	14	145
sul3_v1	0	2	2	4	5	0	3	8	2	0	2	4	1	0	0	1	1	0	1	5	2	7	25
**tetra**																							
tetAB	13	11	15	39	19	12	6	37	9	13	15	37	7	0	2	9	9	6	15	15	0	15	152
tetA	1	7	1	9	11	1	5	17	10	0	2	12	2	3	4	9	2	7	9	6	1	7	63
tetB	13	11	15	39	19	12	6	37	9	13	15	37	7	0	2	9	9	6	15	15	0	15	152
**Trime**																							
dfrA1	0	1	0	1	0	0	2	2	0	0	1	1	1	2	0	3	1	2	3	0	0	0	10
dfrA12	0	2	2	4	4	0	3	7	1	0	2	3	1	0	0	1	1	0	1	5	2	7	23
dfrA14	0	0	1	1	6	0	0	6	3	0	1	4	0	0	0	0	0	1	1	0	0	0	12
dfrA16	0	0	0	0	1	0	0	1	1	0	0	1	0	0	0	0	0	0	0	0	0	0	2
dfrA17	9	6	12	27	4	10	4	18	5	11	11	27	5	0	0	5	4	4	8	3	0	3	88
dfrA5	0	0	0	0	0	0	0	0	0	0	0	0	0	0	0	0	0	1	1	0	0	0	1
**Grand Total**	159	144	206	509	246	150	130	526	165	178	202	545	83	13	17	113	95	112	207	153	40	193	2093

Table S4. Statistical analyses of AMR gene count data.

Data 1	Data 2	Statistical Test	*p*-value
T1 ECC	T1 CIP	T-test, 2 tail, homoscedastic	3.34E-24
T2 ECC	T2 CIP	T-test, 2 tail, homoscedastic	6.69E-27
T3 ECC	T3 CIP	T-test, 2 tail, homoscedastic	2.61E-29
T1 CIP	T2 CIP	T-test, 2 tail, homoscedastic	0.043625
T1 CIP	T3 CIP	T-test, 2 tail, homoscedastic	0.542164
T2 CIP	T3 CIP	T-test, 2 tail, homoscedastic	0.015854

Table S6. Gull isolates identified from time-points 1 and 3 as having clones (<14 SNPs) in pig isolates from one or more time-points. Gull isolates are shown alongside time-point, isolation media, sequence type and total number of pig isolates identified as clonal by time-point.

				Related pig isolates at time-point:		
**Gull Isolate**	**Time Point**	**Media**	**ST**	**T1**	**T2**	**T3**
F1T1-M53	T1	CIP	744	1	0	0
F1T1-M54	T1	CIP	744	4	0	0
F1T1-M56	T1	CIP	744	2	0	0
F1T1-M58	T1	CIP	44	0	2	2
F1T1-M65	T1	CTX	88	6	3	0
F1T1-M66	T1	CTX	88	8	0	0
F1T3-S131*	T3	CIP	744	0	15	14
F1T3-S132	T3	CIP	744	6	3	12
F1T3-S133*	T3	CIP	744	0	10	13
F1T3-S134	T3	CIP	44	0	2	1
F1T3-S135*	T3	CIP	744	0	15	14

Table S7: Annotation of the pIncY-T1M44 and pIncX-T1M17 plasmids shows *tra* and *mob* genes are missing. The pIncX-T1M17 plasmid contains a fim gene but not the full operon.

pIncY-T1M44

Start	End	Strand	Protein
1	861	+	RepB family plasmid replication initiator protein
1255	2466	+	ParA family protein
2463	3428	+	ParB/RepB/Spo0J family partition protein
3917	4633	+	IS6-like element IS26 family transposase
5527	6030	-	IS1 family transposase
6529	7533	+	IS110 family transposase

8209	10299	-	protease Lon-related BREX system protein BrxL
10316	12973	-	BREX-1 system phosphatase PglZ type A
12973	16602	-	BREX-1 system adenine specific DNA-methyltransferase PglX
16618	18381	-	DUF262 domain-containing protein
18398	22075	-	BREX system P-loop protein BrxC
22094	22678	-	DUF1788 domain-containing protein
22675	23274	-	DUF1819 family protein
23284	23985	-	WYL domain-containing protein
24279	24983	-	hypothetical protein
25031	25702	-	IS3 family transposase
27838	28428	+	plasmid pRiA4b ORF-3 family protein
28778	29137	+	resolvase
29171	30565	+	ISKra4-like element ISKpn19 family transposase
30589	31026	+	resolvase
30925	31260	-	hypothetical protein
31345	32001	-	quinolone resistance pentapeptide repeat protein QnrS1
32893	33429	+	IS3 family transposase
33454	36003	-	Tn3-like element Tn3 family transposase
36398	36595	+	cupid fold metalloprotein, WbuC family
36642	37517	-	class A extended-spectrum beta-lactamase CTX-M-15
37687	38553	-	IS3-like element IS3 family transposase
38550	38849	-	IS3 family transposase
39034	39981	-	IS1380-like element ISEc9 family transposase
40477	40854	-	DUF4158 domain-containing protein
40823	41416	+	recombinase family protein
41599	42189	+	class A beta-lactamase TEM-1b
42224	42940	+	IS6-like element IS26 family transposase
43641	44810	+	UDP-N-acetylglucosamine 2-epimerase (non-hydrolizing)
44804	46081	+	UDP-N-acetyl-D-mannosamine dehydrogenase
46119	46865	+	DUF2837 family protein
46894	48111	+	oligosaccharide repeat unit polymerase
48131	49186	+	glycosyltransferase family 1 protein
49330	50955	+	alginate lyase family protein
51213	52181	+	glycosyltransferase WbuB
52367	53269	+	UTP--glucose-1-phosphate uridylyltransferase
53285	53647	+	UDP-glucose 4-epimerase GalE
53682	54398	+	IS6-like element IS26 family transposase
54773	55225	+	hypothetical protein
55241	55843	-	hypothetical protein
55979	57130	+	integrase
57219	57809	+	IS4/IS5 family transposase
57844	58560	+	IS6-like element IS26 family transposase
58506	59618	-	class 1 integron integrase IntI1
59765	60247	+	trimethoprim-resistant dihydrofolate reductase DfrA14
60482	61198	+	IS6-like element IS26 family transposase

61232	61693	-	hypothetical protein
61830	62567	+	resolvase
63268	64272	-	IS110-like element IS5075 family transposase
64351	66057	-	Tn3 family transposase
66346	67752	+	transposase
68084	68761	-	TetR family transcriptional regulator
68765	70039	+	tetracycline efflux MFS transporter Tet(A)
70071	70955	-	EamA family transporter
71444	72199	+	transposase
72338	72574	-	class A beta-lactamase
72685	73401	-	IS6-like element IS26 family transposase
73718	74020	+	XRE family transcriptional regulator
74759	75286	-	nuclease
75506	76459	-	hypothetical protein
76580	77125	-	hypothetical protein
77095	77541	-	Fe3+ siderophore ABC transporter permease
77541	77900	-	hypothetical protein
77958	78386	-	hypothetical protein
78420	79280	-	DsbA family protein
79296	80273	-	S49 family peptidase
80255	81109	-	hypothetical protein
81114	81452	-	hypothetical protein
81418	81861	-	hypothetical protein
81988	82509	-	hypothetical protein
82512	82973	-	hypothetical protein
83456	84655	+	IS91 family transposase

pIncX1-T1M17

Start	End	Strand	Protein
90	3056	-	Tn3-like element TnAs3 family transposase
3059	3619	-	recombinase family protein
3745	4329	-	DUF3330 domain-containing protein
4298	5311	-	class 1 integron integrase IntI1
5456	5953	+	trimethoprim-resistant dihydrofolate reductase DfrA12
6361	7152	+	ANT(3)-Ia family aminoglycoside nucleotidyltransferase AadA2
7501	8673	+	chloramphenicol efflux MFS transporter CmlA1
8766	9557	+	ANT(s)-Ia family aminoglycoside nucleotidyltransferase AadA1
9795	10403	-	IS256 family transposase
10728	11519	-	sulfonamide-resistant dihydropteroate synthase Sul3
12271	13134	-	SDR family oxidoreductase
13280	13534	-	macrolide efflux MFS transporter Mef(B) truncated
13592	14308	-	IS6-like element IS26 family transposase
14382	17348	+	Tn3-like element TnAs1 family transposase
17345	18028	-	TetR family transcriptional regulator
18032	19306	+	tetracycline efflux MFS transporter Tet(A)

19338	20222	-	EamA family transporter
20360	20524	-	isochorismatase family protein
20711	22507	+	Tn3 family transposase
22559	24241	+	fimbrial biogenesis outer membrane usher protein
24254	25012	+	gram-negative pili assembly chaperone domain protein
25014	26078	+	hypothetical protein
26891	27607	+	hypothetical protein
27871	28512	-	restriction endonuclease
28568	28801	-	hypothetical protein
28915	29229	-	KikA protein
29270	29773	-	IS1 transposase
31798	32634	+	RepB family plasmid replication initiator protein
33188	33613	+	putative DNA distortion polypeptide 3
33610	34587	-	hypothetical protein
34612	34920	-	hypothetical protein
34991	35653	-	peptidyl-arginine deiminase
36034	36678	+	resolvase
37141	37644	-	IS1 family transposase
38833	39336	-	IS1 family transposase

Table S8: Results from mobtyper* predicts pIncX1-T1M17 and pIncY-T1M44 to be non-mobilisable plasmids.

sample_id	num_contigs	size	gc	md5	rep_type(s)	rep_type_accession(s)	relaxase_type(s)	relaxase_type_accession(s)	mpf_type	mpf_type_accession(s)	orit_type(s)	orit_accession(s)	predicted_mobility	mash_nearest_neighbor	mash_neighbor_distance	mash_neighbor_identification	primary_cluster_id	secondary_cluster_id	observed_host_range_ncbi_name	associated_pmid(s)
pIncX-T1M17_pk.fna	1	-	51.3831	29c5f8f48bc140e6c795adbc85ab8d96	IncX1	000215__KC853434_00019	-	-	-	-	-	-	non-mobilizable	CP042589	0.010188	Escherichia coli	AB718	AK607	Enterobacterales	21625636; 22470007
pIncY-T1M44_pk.fna	1	-	48.99423	27f81fb33904d98edfa7a751d6cdb70c	IncY	000230__CP009168_00001	-	-	-	-	-	-	non-mobilizable	MF510423	0.016275	Escherichia coli	AC315	AL455	Enterobacteriaceae	28031198
